# Cytokines in Sepsis: Potent Immunoregulators and Potential Therapeutic Targets—An Updated View

**DOI:** 10.1155/2013/165974

**Published:** 2013-06-18

**Authors:** Wibke Schulte, Jürgen Bernhagen, Richard Bucala

**Affiliations:** ^1^Department of Internal Medicine, Yale University School of Medicine, The Anlyan Center, S525, P.O. Box 208031, 300 Cedar Street, New Haven, CT 06520-8031, USA; ^2^Institute of Biochemistry and Molecular Cell Biology, University Hospital of RWTH Aachen University, Pauwelsstraße 30, 52074 Aachen, Germany

## Abstract

Sepsis and septic shock are among the leading causes of death in intensive care units worldwide. Numerous studies on their pathophysiology have revealed an imbalance in the inflammatory network leading to tissue damage, organ failure, and ultimately, death. Cytokines are important pleiotropic regulators of the immune response, which have a crucial role in the complex pathophysiology underlying sepsis. They have both pro- and anti-inflammatory functions and are capable of coordinating effective defense mechanisms against invading pathogens. On the other hand, cytokines may dysregulate the immune response and promote tissue-damaging inflammation. In this review, we address the current knowledge of the actions of pro- and anti-inflammatory cytokines in sepsis pathophysiology as well as how these cytokines and other important immunomodulating agents may be therapeutically targeted to improve the clinical outcome of sepsis.

## 1. Introduction

Sepsis, or the invasion of microbial pathogens into the bloodstream, is characterized by a systemic proinflammatory response, which can lead to severe sepsis and septic shock [1]. Sepsis, severe sepsis, and septic shock are major healthcare problems worldwide; they affect millions of people each year, and their incidence increases annually [2, 3]. Despite significant advances in intensive care treatment over the last years, septic shock remains associated with high mortality rates [4]. An epidemiologic study reported that septic shock is the most common cause of death in noncoronary intensive care units, and the tenth leading cause of death overall in high-income countries [2]. The outcome of sepsis is particularly unfavorable in elderly, immunocompromised, and critically ill patients [5]. Reasons for the anticipated increase in sepsis incidence and its associated mortality include the increasing number of immunocompromised patients, emerging antibiotic resistance in microorganisms, and the aging population [6].

 Besides its clinical challenge, the treatment of sepsis imposes a large economic burden on healthcare systems worldwide [7]. With an estimated 750,000 cases occurring in the United States alone each year, the annual total costs have been estimated to be approximately $16.7 billion nationally [8]. Sepsis was identified as one of the five conditions that account for the most expensive hospital stays in the United States [7].

## 2. Definition of Sepsis

The word “sepsis” is derived from the word “*σηψις*,” which in the original Greek means “decomposition” or “putrefaction,” and was first mentioned in Homer's poems approximately 2700 years ago [9]. Only relatively recently have studies led to detailed descriptions of the clinical findings in septic patients and to an understanding of the underlying pathophysiology. These findings in turn have led to redefinitions of sepsis and its sequelae. Generally, sepsis is viewed as the response of the host toward invading pathogens or its toxins and is a syndrome that consists of multiple clinical and biochemical findings [10]. In 1991, a consensus conference was held by the American College of Chest Physicians (ACCP) and the Society of Critical Care Medicine (SCCM) to develop a single and universally accepted definition of sepsis to improve the early diagnosis and treatment of the disease and facilitate research. A key result of this consensus conference was the introduction of the term “systemic inflammatory response syndrome” (SIRS) which was defined as a combination of clinical signs without the existence of an underlying infection [1, 11] (Table 1). SIRS can be triggered by a variety of noninfectious conditions, such as trauma, burns, hemorrhagic or hypovolemic shock, pancreatitis, and other disease states. In contrast, the diagnosis of sepsis requires clinical evidence of infection along with an underlying SIRS disease state. Severe sepsis is characterized as sepsis complicated by acute organ dysfunction, hypoperfusion, or hypotension [1]. It may lead to “multiple organ dysfunction syndrome” (MODS), or septic shock. Septic shock refers to a state of acute circulatory failure that is characterized by persistent arterial hypotension (systolic pressure <90 mmHg or a mean arterial pressure <60 mmHg) despite adequate fluid resuscitation and in the absence of other causes of hypotension [1].

 Following the 1991 consensus conference, the SIRS criteria were rapidly adopted by many clinicians and scientists and were widely used to select patients for clinical trials. However, many authors criticized the SIRS diagnostic criteria for their poor specificity and lack of prognostic value, as these criteria are broad and limited in number [12–14]. In 2001, an International Sepsis Definition Conference convened aiming to evaluate the previous definitions of SIRS, sepsis, severe sepsis, and septic shock [11]. Following this conference, an expanded list of clinical and biochemical diagnostic criteria for sepsis was released, which better reflected this complex disease state. In 2004, a committee of international sepsis experts published clinical practice guidelines for the management of severe sepsis and septic shock [15]. These guidelines were widely disseminated as part of the “Surviving Sepsis Campaign” and are regularly updated, with the last revision made in 2013 [10].

## 3. Pathophysiology of Sepsis

In recent years, a significant body of literature has been published in an attempt to understand the complex and dynamic pathophysiologic mechanisms that underlie the heterogeneous sepsis syndrome. Sepsis has been shown to develop when the initial, appropriate host response to an infection becomes amplified and subsequently dysregulated [16], leading to an imbalance between proinflammatory and anti-inflammatory responses. It has been reported that the innate immune response, which unlike the adaptive immune response, is able to immediately respond to invading pathogens, plays a major role in the initiation of sepsis pathophysiology [17]. The activation of this “first line of cellular defense” results in an excessive release of cytokines, chemokines, and other inflammatory regulators. Cytokines regulate a variety of inflammatory responses, including the migration of immune cells to the locus of infection, which is a crucial step in containing a localized infection and preventing it from becoming systemic. However, a dysregulated cytokine release may lead to endothelial dysfunction, characterized by vasodilation and increased capillary permeability. The resulting leakage syndrome is clinically associated with hypotension, hemoconcentration, macromolecular extravasation, and edema, which are frequent findings in septic patients [18]. The dysfunctional epithelial barriers enable pathogens and their products to further invade the host organism, to disturb regulatory mechanisms, and ultimately, to cause remote organ dysfunctions [19]. Moreover, increasing evidence has indicated that immune and inflammatory responses are tightly interwoven with different physiologic processes within the human host, such as coagulation [20], metabolism [21, 22], and neuroendocrine activation [23, 24]. An inflammation-induced dysregulation of the coagulation system, for instance, significantly aggravates the deleterious effects of sepsis and can result in lethal disseminated intravascular coagulation [25].

Traditionally, sepsis was viewed as an excessive systemic proinflammatory reaction to invasive microbial pathogens. More recently, it has been proposed that the early phase of hyperinflammation is followed or overlapped by a prolonged state of immunosuppression [26–28], referred to as sepsis-induced immunoparalysis [29]. This immunoparalytic state is characterized by impaired innate and adaptive immune responses and, may play a central role in the pathogenesis of tissue damage, multiple organ failure, and death induced by sepsis. 

## 4. Initiation of the Immune Response

**Figure 1:**
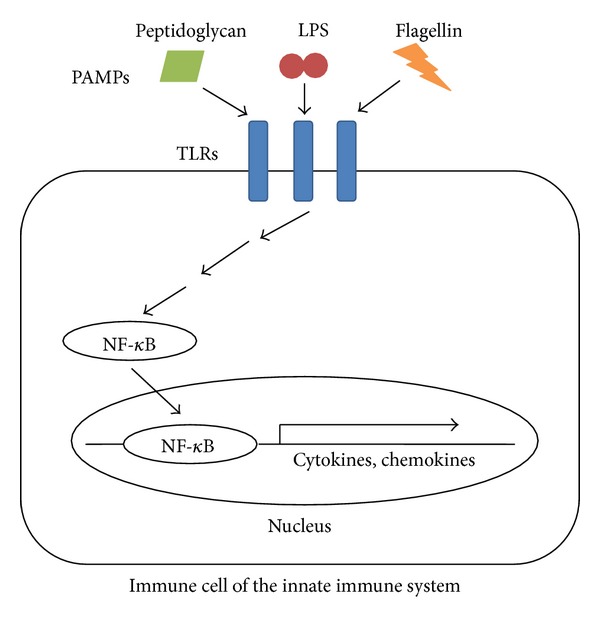
Initiation of the immune response following infection. Immune cells of the innate immune system recognize invading pathogens via Toll-like receptors (TLRs). The binding of pathogen-associated molecular patterns (PAMPs), such as peptidoglycan, lipopolysaccharide (LPS), or flagellin, to TLRs initiates signal transduction cascades that lead to the activation of nuclear factor *κ*B (NF-*κ*B). NF-*κ*B is subsequently translocated into the nucleus where it induces the expression of cytokines and chemokines.

The innate immune system detects invading microorganisms via pathogen recognition receptors (PRRs), which are expressed on epithelial barriers as well as on immune cells such as dendritic cells and macrophages [30] (Figure 1). A specific family of PRRs named Toll-like receptors (TLRs) recognizes conserved macromolecular motives from microorganisms, called pathogen-associated molecular patterns (PAMPs). Examples of bacterial PAMPs include lipopolysaccharide (LPS; the main virulence factor of Gram-negative bacteria), peptidoglycan, lipoteichoic acid (a cell wall component of Gram-positive bacteria), flagellin, and bacterial DNA [6, 31]. The stimulation of TLRs or the NOD-like receptor (NLR) family of intracellular PRRs results in the triggering of downstream signaling cascades. Depending on the particular receptor engaged, this process leads to the activation of a transcriptional response program that includes nuclear factor *κ*B (NF-*κ*B), followed by the production and secretion of cytokines, chemokines, and nitric oxide (NO) [32–34].

## 5. Cytokines in Sepsis Pathophysiology

The term cytokine describes a functional class of small protein mediators with low molecular weights (mostly <40 kDa), which are produced in a regulated fashion to affect the activation and differentiation of the immune response. Once released, proinflammatory cytokines lead to an ensuing activation of the innate or the adaptive immune response, characterized by the further production of immunoregulatory or effector cytokines [97]. The sequential release of specific cytokines is referred to as a “cytokine cascade” [98]. In the 1990s, sepsis was believed to be associated with an exacerbated release of mainly proinflammatory cytokines, such as tumor necrosis factor (TNF)-*α*, interleukin (IL)-1, IL-6, IL-12, interferon (IFN)-*γ*, and macrophage migration inhibitory factor (MIF). The term “cytokine storm” thus arose [99]. However, recent research on the pathophysiologic mechanisms underlying sepsis indicates that the profound proinflammatory response is counteracted by certain anti-inflammatory cytokines, including IL-10, transforming growth factor (TGF)-*β*, and IL-4, which attempt to restore immunological equilibrium [16, 100]. Lately, efforts have been made to identify unifying mechanisms by employing genome-wide expression data in early and late sepsis. Tang et al. reported that sepsis leads to the immediate upregulation of PRRs and the activation of signal transduction cascades [101]. However, important inflammatory markers, such as TNF-*α*, IL-1, or IL-10, did not show any consistent pattern in their gene expression and are highly variable in individuals. These findings suggest that the host response to sepsis is not a simple model with an initial proinflammatory phase followed by an anti-inflammatory response, but rather a highly interactive and dynamic process that may reflect heterogeneous genome-specific pathways. A tightly regulated balance in the cytokine network, which comprises proinflammatory cytokines, anti-inflammatory cytokines, and soluble inhibitors of proinflammatory cytokines, such as soluble TNF receptors (sTNFRs), IL-1 receptor antagonist (IL-1Ra), and IL-1 receptor type II (IL-1R2), is crucial for eliminating invading pathogens on the one hand and restricting excessive, tissue-damaging inflammation on the other [102, 103].

 This review summarizes current knowledge of the role of cytokines in the regulation of the immune response in sepsis. The actions of individual pro- and anti-inflammatory cytokines are described in more detail and are directly associated with sepsis pathophysiology (see Table 2 for a summary). Along with the increasing knowledge of cytokine actions in recent years, a number of therapeutic strategies targeting cytokines and other immunomodulating agents have been proposed for clinical use in septic patients. Their current role in the treatment of sepsis is discussed later in this review.

## 6. Proinflammatory Cytokines

### 6.1. TNF-*α* and IL-1

TNF-*α* and IL-1 (a term used for a family of proteins, including IL-1*α* and IL-1*β* [104]) are among the most extensively studied cytokines in sepsis pathophysiology. Both are powerful proinflammatory cytokines that have been implicated in a large number of infectious and noninfectious inflammatory diseases, the latter including atherosclerosis [35], rheumatoid arthritis [36], osteoarthritis [105], and Alzheimer's disease [38]. TNF-*α* is a 17 kDa protein that is not only derived predominantly from activated immune cells (macrophages) but also from nonimmune cells (fibroblasts) in response to invasive, infectious, or inflammatory stimuli [37, 40]. The release of TNF-*α* from macrophages begins within 30 minutes after the inciting event, following gene transcription and RNA translation, which established this mediator to be an early regulator of the immune response. TNF-*α* acts via specific transmembrane receptors, TNF receptor (TNFR)1, and TNFR2 [106], leading to the activation of immune cells and the release of an array of downstream immunoregulatory mediators. Likewise, IL-1 is released primarily from activated macrophages in a timely manner similar to TNF-*α*, signals through two distinct receptors, termed IL-1 receptor type I (IL-1R1) and IL-1R2, and has comparable downstream effects on immune cells [44, 47]. The injection of TNF-*α* into experimental animals causes a syndrome that is largely indistinguishable from septic shock [107] and infusion of recombinant TNF-*α* into humans results in SIRS [108–110]. Similar results were reported for IL-1 [111–113]. TNF-*α* and IL-1 act synergistically to induce a shock-like state characterized by vascular permeability, severe pulmonary edema, and hemorrhage [113]. Importantly, TNF-*α* and IL-1 were also identified as pivotal mediators for the development of fever and, thus, belong to a group of pyrogenic cytokines [39].

A role for TNF-*α* and IL-1 in sepsis was demonstrated in numerous reports, including both experimental animal models of septic shock and studies in humans with sepsis. The administration of bacterial endotoxin results in the production and release of TNF-*α* and IL-1 into the systemic circulation, where peak concentrations are detected 60–90 min after LPS administration [114–117]. Once released, TNF-*α* and IL-1 act on different target cells, such as macrophages, endothelial cells, and neutrophils. TNF-*α* leads to an enhanced production of macrophages from progenitor cells [118], promotes the activation and differentiation of macrophages [43], and prolongs their survival [119]. All these effects enhance proinflammatory responses in sepsis. In endothelial cells, TNF-*α* enhances the expression of adhesion molecules, such as intercellular adhesion molecule (ICAM)-1 and vascular cell adhesion molecule (VCAM)-1, and chemokines [120, 121]. TNF-*α* also increases integrin adhesiveness in neutrophils and promotes their extravasation into tissues. TNF-*α* and IL-1 were identified as the main mediators of inflammation-induced activation of coagulation, with TNF-*α* having a potent upregulating action on endothelial expression of procoagulant [42]. In addition, TNF-*α* and IL-1 amplify inflammatory cascades in an autocrine and paracrine manner by activating macrophages to secrete other proinflammatory cytokines (IL-6, IL-8, and MIF), lipid mediators, and reactive oxygen and nitrogen species [16, 46], leading to sepsis-induced organ dysfunction. Because of its unique ability to orchestrate downstream cytokine cascade, TNF-*α* is considered to be a “master regulator” of inflammatory cytokine production [37], while the important regulatory role of IL-1 in inflammation is widely accepted as well.

Soluble cytokine receptors and receptor antagonists, termed sTNFRs, IL-1R2, and IL-1Ra, were identified for TNF-*α* and IL-1, which modulate the actions of these cytokines. Elevated levels of sTNFRs and IL-1Ra were measured in the systemic circulation of healthy volunteers administered endotoxin [122, 123], and in septic patients, in whom sTNFRs and IL-1Ra plasma concentrations also correlated with disease severity, and in the case of sTNFRs, with mortality [124–126]. In different murine models of septic shock, the administration of IL-1Ra increased survival, suggesting a therapeutic effect for IL-1Ra [48, 127]. For sTNFRs, it was proposed that the ratio between TNF-*α* and sTNFRs, rather than the absolute plasma concentration of TNF-*α* or sTNFRs alone, has prognostic value in septic patients [41]. This indicates that a tight balance between cytokines and their soluble inhibitors is crucial for a positive outcome of sepsis. However, the exact mechanisms underlying this balance remain incompletely understood.

### 6.2. IL-6

IL-6 is a 21 kDa glycoprotein produced by a wide variety of cells, especially macrophages, dendritic cells, lymphocytes, endothelial cells, fibroblasts, and smooth muscle cells in response to stimulation with LPS, IL-1, and TNF-*α* [53–56]. Elevated IL-6 concentrations are measured in many acute conditions, such as burns, major surgery and sepsis [52], and peak subsequent to TNF-*α* and IL-1 concentrations [123, 128]. Plasma levels of IL-6 are stably elevated in these conditions and correlate with many indicators of disease severity such as clinical scores [129], stress after surgery [130] and trauma [131], the occurrence of multiple organ failure and septic shock [132, 133], and the overall mortality [134].

IL-6 has a variety of biological effects, including the activation of B and T lymphocytes and the coagulation system, and the modulation of hematopoiesis [49, 61]. In contrast to TNF-*α* and IL-1, the injection of IL-6 by itself does not produce a sepsis-like state [135]. A key function of IL-6 is the induction of fever [50] and the mediation of the acute phase response [51, 59], a systemic reaction to an inflammatory stimulus that is characterized by fever, leukocytosis, and the release of hepatic acute phase proteins such as C-reactive protein, complement components, fibrinogen, and ferritin [136]. *In vivo* studies in *Il-6*-knockout mice demonstrated that the deletion of the *Il-6* gene decreases lung inflammation in a model of acute lung injury [137] and protects from mortality and the development of organ failure in a zymosan-induced acute peritoneal inflammation [138]. More recently, Pathan et al. showed that IL-6 causes myocardial depression in meningococcal disease [139]. Myocardial dysfunction in septic shock leads to impaired tissue perfusion, multiorgan failure, and death.

Despite its proinflammatory properties, IL-6 also has been shown to promote anti-inflammatory responses. IL-6 inhibits the release of TNF-*α* and IL-1 [57] and enhances the circulation levels of anti-inflammatory mediators, such as IL-1Ra, sTNFRs, IL-10, TGF-*β*, and cortisol [58, 60, 62]. A protective effect of IL-6 was shown in experimental endotoxemia [140, 141], whereas the genetic deletion of IL-6 did not alter the mortality in a model of polymicrobial sepsis induced by cecal ligation and puncture (CLP) [142].

### 6.3. IL-12

Phagocytes (monocytes/macrophages and neutrophils) and dendritic cells are the major sources of the heterodimeric cytokine IL-12 [66, 67], which is structurally related to the IL-6 cytokine family [143]. IL-12 regulates innate immune responses and promotes the development of a type 1 adaptive immune response, which is characterized by enhanced mononuclear phagocyte responses. Thus, IL-12 links early, nonspecific, and later, specific immune responses. Upon release, IL-12 induces T-cells and natural killer (NK) cells to produce IFN-*γ*, which directly activates macrophages to enhance their bactericidal activity and produce additional T helper 1 (T_H_1) cytokines [64]. Additionally, IL-12 stimulates the differentiation of naive CD4^+^ T-cells into T_H_1 cells and protects them from antigen-induced apoptotic death [65]. IL-12 also increases the proliferation and colony formation of hematopoietic progenitors.

 Despite many years of research, the role of IL-12 in sepsis remains controversial. Initially, animal models of sepsis were employed to investigate its role in sepsis. Increased plasma IL-12 concentrations were measured in animals following the administration of LPS or *Escherichia coli*, and after polymicrobial sepsis induced by CLP [144–146]. The immunoneutralization or genetic deletion of IL-12 resulted in an increased mortality of mice undergoing CLP, with a subsequent decrease in IFN-*γ* and an increase in IL-10 levels [144, 147]. However, in a different animal model, an increase in LPS-induced mortality was observed in mice transiently overexpressing IL-12 and the neutralization of IL-12 improved survival following LPS challenge [145]. Clinically, a prospective study in patients undergoing major visceral surgery suggested that a selective defect in preoperative monocyte IL-12 production impairs the host defense against postoperative infections and, thus, increases the risk of lethal sepsis [148]. Likewise, it was reported that survivors from severe sepsis produce more IL-12 from LPS-stimulated peripheral blood mononuclear cells (PBMCs) than nonsurvivors [149], and that they show serial increases in their IL-12 response from PBMCs [150].

### 6.4. IFN-*γ*

IFN-*γ* is mainly produced by activated NK cells, T_H_1, and CD8^+^ cytotoxic T cells [68]. Its production is tightly regulated and stimulated by macrophage-derived cytokines, especially TNF-*α*, IL-12, and IL-18 [72]. IFN-*γ* was discovered due to its antiviral activity [73]. Subsequently, the important immunoregulatory role of IFN-*γ* to a wider range of pathogens became evident. Mice lacking IFN-*γ* were shown to be more susceptible to intracellular pathogens, such as *Leishmania major* [151], *Listeria monocytogenes* [152], Mycobacteria [153], and different viruses [154]. The neutralization of IFN-*γ* or its receptor makes mice more resistant to an LPS-induced shock [155, 156]. IFN-*γ* is normally not detectable in the plasma of healthy humans, but its levels can be elevated in patients with sepsis [69]. Plasma levels of IFN-*γ* do not correlate with sepsis severity or mortality. Recently, a role for IFN-*γ* in the reversal of sepsis-induced immunoparalysis was reported. During the immunoparalytic state, macrophages were shown to display impaired phagocyte functions and to release reduced amounts of T_H_1-promoting cytokines upon stimulation with bacterial products [157, 158]. Flohé et al. showed that IFN-*γ*, as well as granulocyte-macrophage colony-stimulating factor (GM-CSF), was able to restore macrophage function in macrophages taken from septic mice upon bacterial stimulation *ex vivo* [70]. Likewise, a recently published *in vivo* study in humans demonstrated that IFN-*γ* partially reverses immunoparalysis, identifying IFN-*γ* as a potential new treatment option for sepsis [71].

### 6.5. MIF

MIF is a pleiotropic proinflammatory cytokine, which is responsible for the first cytokine activity to be discovered [159]. MIF is released by pituitary cells in response to LPS and stress [74, 77] and by immune cells (most importantly monocytes and macrophages) after exposure to various infectious and inflammatory stimuli, including LPS, TNF-*α*, and IFN-*γ* [78, 160]. Uniquely among innate cytokines, MIF is present in preformed pools within cells and is rapidly released upon proinflammatory and stress stimulation [161]. This release response of the preformed protein is followed by MIF gene transcription and RNA translation, which replenishes intracellular stores. The Golgi complex-associated protein p115 was identified as an intracellular binding partner for MIF that is essential for its secretion [161]. Once secreted, MIF increases macrophage antimicrobial responses by increasing macrophage survival [83], elevating TLR4 expression on macrophages [162] and promoting macrophage inflammatory recruitment [75]. MIF also promotes the secretion of downstream cytokines, such as TNF-*α*, IFN-*γ*, and IL-1, and it promotes the activation of T cells [80]. MIF activates immune cells by binding to CD74 (the cell surface form of the class II-associated invariant chain), which leads to the recruitment of CD44 into a signaling complex and the downstream initiation of the ERK1/2 MAP kinase pathway [163, 164]. Additionally, MIF engages the chemokine receptors CXCR2 and CXCR4 in a high affinity, noncognate interaction [75]. While the precise signaling mechanisms of MIF through these receptors are yet to be clarified, it was demonstrated that the MIF/CXCR axis is critical for MIF-dependent monocyte recruitment processes in atherosclerotic arteries [75]. MIF's critical role within the immune system is further underscored by the finding that MIF is induced by low concentrations of glucocorticoids and has the unique ability to override the anti-inflammatory and immunosuppressive effects of glucocorticoids [77, 81, 84]. Mouse modeling and human clinical studies have implicated MIF in the pathogenesis of various acute and chronic inflammatory diseases, including septic shock [79], asthma [165], rheumatoid arthritis [166], atherosclerosis [167], inflammatory bowel disease [168], and cancer [76].

 The actions of MIF in sepsis pathophysiology have been studied extensively. The administration of recombinant MIF protein increases mortality following LPS administration [74]. Conversely, several studies showed that the neutralization of MIF reduced proinflammatory cytokine production, decreased organ injury, and increased the survival rate of mice in different animal models of sepsis, such as endotoxic shock, *Escherichia coli* injection or CLP [79, 169–172]. Recently, MIF was established as an important mediator of LPS-induced myocardial dysfunction [173, 174]. Serum MIF concentration of patients suffering from sepsis are significantly higher compared to healthy individuals [175] and correlate with the outcome [176]. Thus, MIF was suggested as an early predictor for survival in septic patients [177]. In the largest genetic study of sepsis performed to date, *MIF* alone among 20 candidate polymorphic loci within immune response genes was associated with clinical outcome from septic shock [178]. Notably, the role for *MIF* gene variants in this study of community-acquired pneumonia progressing to sepsis was found to be one of protection, with a 50% survival benefit observed in individuals with high expression *MIF* alleles at 30, 60, and 90 days of followup. Thus, despite prior suggestions that sepsis pathology results from an excessive or overreactive systemic inflammatory response, high MIF expression was protective, presumably because of its high upstream role in eliminating invasive microbial infections or because of its ability to counteract the immunoparalytic state. A strong role for MIF also has been reported for clinical outcome from meningococcemia [179], invasive streptococcal infection [180], and severe malaria [181].

 Very recently, the protein D-dopachrome tautomerase (D-DT), which is the only known MIF homolog in the human genome, was identified as a cytokine [82, 175]. While the precise biologic functions of D-DT (a.k.a. MIF-2) are yet to be clarified, it was demonstrated that D-DT is released in response to LPS and that its immunoneutralization protects mice from lethal endotoxic shock. This protective action of anti-D-DT was associated with a reduction in the circulating levels of TNF-*α*, IFN-*γ*, IL-12, and IL-1 and increases in the serum concentration of IL-10. D-DT serum levels have been determined to be higher in septic patients compared to healthy controls and to correlate with MIF and with disease severity.

## 7. Anti-Inflammatory Cytokines

### 7.1. IL-10

IL-10 is a 35-kDa homodimeric cytokine that is produced by many types of immune cells, such as monocytes, macrophages, B and T lymphocytes, and NK cells [87]. Functional studies widely revealed anti-inflammatory functions of IL-10. *In vitro*, IL-10 suppresses the production of proinflammatory mediators, such as TNF-*α*, IL-1, IL-6, IFN-*γ*, and GM-CSF, in immune cells [85, 86]. In contrast, it was reported that IL-10 has no effect on the constitutive expression of TGF-*β*, a cytokine with anti-inflammatory properties. Additionally, IL-10 stimulates the production of IL-1Ra and sTNFRs, thereby neutralizing the proinflammatory actions of IL-1 and TNF [88]. These results were supported *in vivo*. In an experimental murine model, the administration of recombinant IL-10 protein protected mice from lethal endotoxemia, even when IL-10 was injected 30 minutes after the LPS administration [182]. In contrast, the immunoneutralization of IL-10 led to elevated levels of circulating TNF-*α* and IL-6 in mice [183] and reversed the ability of IL-10 to protect mice from lethal endotoxemia [182]. Despite these clearly protective effects of IL-10 in LPS-induced pathologies, the actions of IL-10 were not always beneficial in the CLP model of polymicrobial sepsis. In fact, the inhibition of IL-10 12 hours after CLP markedly improved survival [184]. However, the administration of neutralizing IL-10 antibodies at the time of CLP partially exacerbated mortality [185]. These findings indicate that the time of anti-IL-10 antibody application is crucial for the outcome, and that IL-10 can exhibit protective or harmful effects in the course of sepsis. More recently, Latifi et al. reported that IL-10-deficient mice showed an earlier onset of lethality following CLP and showed a reduced response to rescue surgery (the removal of the necrotic cecum) compared with wildtype mice [87]. However, the administration of recombinant IL-10 protein to WT or IL-10 deficient mice increased survival and lengthened the therapeutic window for the rescue surgery. These results suggest that IL-10 might regulate the transition from early reversible sepsis to late irreversible septic shock. Recently, it was investigated whether polymorphisms in the IL-10 gene promotor affect sepsis susceptibility. Zeng et al. showed that the −1082A allele was associated with a lower IL-10 production following LPS stimulation and with the development of sepsis after major trauma [186].

### 7.2. TGF-*β*

TGF-*β* is a member of a family of dimeric polypeptide growth factors and is an important anti-inflammatory cytokine. A role for TGF-*β* was demonstrated in tissue repair und fibrosis [93], as well as in sepsis-induced immunosuppression [89]. *In vitro*, TGF-*β* suppresses the release of proinflammatory mediators, such as IL-1, TNF-*α*, and HMGB1, from monocytes and macrophages [90, 92], and stimulates the production of immunosuppressive factors such as sTNFRs and IL-1Ra [94]. TGF-*β* also inhibits T lymphocyte functions, such as IL-2 secretion and T cell proliferation [187], and it promotes the development of T regulatory cells [188]. Moreover, studies demonstrated a role of TGF-*β*, as well as IL-10, in the tolerance of monocytes and macrophages to LPS, which is characterized by a downregulated cytokine response following a second LPS challenge [189].

In alignment with the *in vitro* studies, experiments in animal models of sepsis and clinical studies in humans supported the anti-inflammatory actions of TGF-*β*. Parrella et al. reported that treatment with TGF-*β* blocked endotoxin-induced hypotension, potentially by inhibiting the hypotensive effects of NO and improved survival in a rat model of *Salmonella typhosa* endotoxin-induced septic shock [190]. Similar results were reported in a rat model using the endotoxin of *Salmonella enteritidis* [191] and in the murine endotoxic shock model [192]. Moreover, patients with sepsis had elevated levels of TGF-*β* compared to healthy controls [91]. TGF-*β* levels were shown to peak early in disease progression and not to correlate strongly with disease severity or prognosis [193]. Recent data demonstrated that TGF-*β* reverses the depression of cardiac myocyte contraction, which is induced by proinflammatory cytokines, such as TNF-*α* and IL-1, and by serum from patients with septic shock [194]. This suggests that TGF-*β* might have cardio-protective effects in sepsis-induced cardiac injury.

### 7.3. IL-4

IL-4 is a cytokine with many immunoregulatory functions, which was shown to participate in the regulation of proliferation, differentiation, and apoptosis of multiple cell types [195–197]. An important action of IL-4 is its critical role in the regulation of T lymphocyte differentiation, in which it promotes T_H_2 cell differentiation while inhibiting T_H_1 cell differentiation [96]. IL-4 is the principal cytokine produced by T_H_2 lymphocytes, causes an enhanced release of further IL-4 and other anti-inflammatory cytokines, and suppresses the secretion of monocyte-derived proinflammatory cytokines [95].

 Animal-based studies revealed that IL-4 increases survival of mice exposed to lethal doses of LPS [198]. However, protective as well as detrimental effects of IL-4 were described in *Staphylococcus aureus*-triggered murine sepsis, which appeared to depend on the host's genetic background [199]. In humans, it was reported that the mRNA expression of IL-4 was associated with survival of patients with severe sepsis, but that the plasma IL-4 levels in septic patients on the day of admission to the hospital did not differ between survivors and nonsurvivors [200]. Recently, it was suggested that *IL-4* promoter polymorphisms might affect the balance between the T_H_1 and T_H_2 immune response, and thereby predispose trauma patients to the development of sepsis [201]. While all these studies indicate that IL-4 plays an important role in the pathogenesis of sepsis, its precise role during the course of disease remains unknown.

## 8. Immunomodulating Treatment Strategies for Sepsis

Basic research and clinical studies performed over the past several years have led to a significant amount of data on immunoregulatory and modulating mechanisms in sepsis. Cytokines have proved to function as important regulators of the immune response, while various other agents, including growth factors or activated protein C (APC), have shown immunomodulating effects. Therefore, it would appear to be highly promising and beneficial to therapeutically target these mediators in order to decrease the unfavorable effects of sepsis-related host responses, and to improve the overall outcome. A number of potential therapeutic targets have been identified to date, and their clinical use has subsequently been assessed in sepsis, both in animal models and in clinical trials. The following paragraphs will give an overview of recent important therapeutic strategies for the treatment of sepsis with special respect to anticytokine approaches.

### 8.1. Anti-TNF-*α* and Anti-IL-1

In one of the first approaches of treating sepsis, therapies were directed against TNF-*α* and IL-1. These therapies included monoclonal antibodies against TNF-*α* [202], sTNFRs [45], IL-1Ra, and soluble IL-1 receptors [203]. While positive results were obtained in experimental models of sepsis, these agents failed to decrease the overall mortality of septic patients in clinical trials [204, 205]. Theses clinical results were unexpected, as the powerful cytokines TNF-*α* and IL-1 had been shown to initiate the excessive inflammatory immune response in sepsis, which was believed to cause the deleterious effects on the host organism. Subsequent studies were conducted to explain the lack of success of TNF-*α* and IL-1 blocking agents in clinical trials. Among many potential reasons, it was reported that the circulating levels of “early” cytokines like TNF-*α* and IL-1 return to almost baseline levels within the first few hours during the progression of disease [206]. Thus, the specific inhibition of “early” cytokines may provide only a narrow window for clinical intervention. Moreover, the elevation of their circulating levels may be downregulated even before the diagnosis of sepsis is made [207], indicating that the early diagnosis of sepsis is crucial for the outcome. It was proposed that inhibiting cytokines like MIF, whose immunoneutralization protected mice from lethal peritonitis even when the antibodies were administered after the onset of disease [79], or HMGB1, which may be involved in later stages of sepsis, might be beneficial in reducing sepsis mortality.

### 8.2. Anti-MIF

Given the complex role of MIF in various pathologies, such as sepsis, MIF is under investigation as a target for the development of novel pharmacological agents. Crystallographic studies of human MIF have identified a tautomerase enzymatic activity site that is important for MIF's cytokine activities [169, 208]. This offers the unique possibility to target a cytokine by a small molecule approach. In fact, small molecules like ISO-1 [(S,R)-3-(4-hydroxyphenyl)-4,5-dihydro-5-isoxazole acetic acid methyl ester] were found to inhibit this catalytic site and to block MIF interaction with its receptor [209] and its downstream effects. ISO-1 suppresses the MIF-induced activation of NF-*κ*B (although NF-*κ*B has so far not emerged as the predominant pathway induced by MIF) and the MIF-induced production of TNF-*α* from macrophages *in vitro* [169]. *In vivo*, the administration of ISO-1 dose-dependently improves survival in a murine model of lethal endotoxemia and rescues mice from polymicrobial sepsis, even when the ISO-1 treatment is started 24 hours after the CLP surgery [169]. ISO-1 or anti-MIF monoclonal antibody administration also was beneficial in a model of lethal flavivirus infection [210]. The positive results obtained from animal models have helped to prompt the clinical development of specific MIF blocking agents. Currently, a human anti-MIF antibody is in clinical development [211], and small molecule MIF inhibitors such as potent benzoxazol-2-ones are advancing towards clinical application [209, 212]. MIF-derived peptide sequences targeting MIF/receptor interfaces also have been considered as potential strategies [213].

### 8.3. IFN-*γ*- and GM-CSF-Directed Strategies

In light of recent research indicating that an immunosuppressive state may contribute to sepsis pathophysiology, it may be advantageous to apply IFN-*γ* or growth factors, such as GM-CSF, in order to restore the host immune functions. Clinical studies showed that GM-CSF improved the gas exchange in patients with severe sepsis associated with respiratory dysfunction [214] and resulted in a more effective anti-infectious defense [215]. However, in neither study did treatment with GM-CSF improve mortality. Also, IFN-*γ* given intravenously to severely injured patients was not successful in decreasing infection rates or improving survival [216].

### 8.4. APC-Directed Strategies

Numerous studies have revealed functional interactions between inflammation and coagulation that contribute significantly to sepsis pathophysiology [20, 217]. Inflammation mediates the coagulation cascade, leading in the extreme case to the development of disseminated intravascular coagulation, and clotting factors in return reciprocally modulate the local or the systemic inflammatory response. Therefore, therapeutic intervention in the coagulation pathway might not only counteract the deleterious effects attributed to a dysregulated coagulation system but also affect the dysregulated inflammatory and immune response in a beneficial manner.

Recombinant human activated protein C (rhAPC) was the first biological drug for the treatment of sepsis that was approved by the Food and Drug Administration in the United States. Protein C is produced by the liver as an acute phase zymogen and is subsequently activated by thrombin [218]. Upon its activation, APC proteolytically inactivates factors Va and VIIIa of the coagulation cascade, resulting in a decrease in thrombin production. Low thrombin levels ultimately lead to the inhibition of the thrombin-induced platelet activation. These anticoagulant actions of APC were considered initially to be responsible for its beneficial effect in sepsis. However, more recent studies have suggested an additional anti-inflammatory action of APC. By preventing the excessive generation of thrombin, APC reduces thrombin's strong proinflammatory actions [219], which include the release of chemokines and cytokines (such as MIF) and the expression of adhesion molecules on platelets and endothelium [220]. Moreover, APC was shown to inhibit chemotaxis and IL-6 release by human neutrophils [221] and to prevent the production of proinflammatory cytokines, such as TNF-*α*, IL-1**β**, IL-6, MIF, and IL-8 by LPS-stimulated monocytes [222, 223]. Antiapoptotic functions have also been attributed to APC. Bilbault et al. showed that circulating mononuclear cells from septic shock patients treated with rhAPC had decreased Bax/Bcl-2 protein ratios compared to healthy controls [224]. Low Bax/Bcl-2 protein ratios are found in antiapoptotic states, which might be beneficial in the recovery from sepsis as high apoptotic rates of immune cells were shown to contribute to the immunoparalytic state of sepsis [225].

Following preclinical investigations of septic shock showing that the administration of APC improved survival [226], the first reports describing the impact of rhAPC administration in humans with severe sepsis were published in 2001. Bernard et al. reported that a 96-hour continuous infusion of rhAPC, also referred to as drotrecogin alfa (activated) (DrotAA), markedly reduced the circulating levels of D-dimers (fibrin degradation products) and IL-6 in patients with severe sepsis [227]. The PROWESS phase 3 clinical trial subsequently showed that the treatment of severe sepsis with rhAPC reduced the relative and absolute death risk by 19.4 and 6.1%, respectively [228]. However, an increased incidence of serious bleeding events was observed in the rhAPC-treated group compared to the placebo group. Nonetheless, on the strength of the survival results, rhAPC was approved for clinical use. Because subgroup analysis in the PROWESS study showed that reduced mortality in the rhAPC-treated group was limited to patients with high disease severity, for instance, those with at least two sepsis-induced dysfunctional organs or those with a high acute physiology and chronic health evaluation (APACHE) II score, the international guidelines for the management of severe sepsis, and septic shock released in 2008 recommended that rhAPC only be used for patients at high risk of death [229]. Unfortunately, more recently published results from the follow-up PROWESS-SHOCK trial indicated that rhAPC did not significantly reduce mortality of patients with septic shock. In fact, at both 28 and 90 days after the initiation of treatment, there was no significant difference in the mortality rate between septic patients treated with rhAPC and those given a placebo (26.4 versus 24.2% and 34.1 versus 32.7%, resp.) [230]. These results have now led to the withdrawal of rhAPC from the market. Future studies will be required to clarify whether rhAPC ultimately finds clinical utility, perhaps in a carefully defined subset of subjects with sepsis. Current treatment modalities for sepsis remain largely supportive rather than directly immunomodulating.

## 9. Conclusion

Sepsis remains a major challenge both for clinicians and researchers. Despite many years of intensive research and numerous clinical studies, its pathophysiology is still incompletely understood, and specific anticytokine treatments have not been successful in clinical trials. This is mainly due to the fact that sepsis can be characterized as a complex and dynamic disease process that involves excessive and suppressed inflammatory and immune responses. Moreover, it affects heterogeneous patient populations with diverse disease etiologies and comorbidities, further aggravating our difficulties in understanding and therapeutically intervening in this complex syndrome. Nonetheless, research studies have elucidated many different pathophysiologic processes involved in sepsis and have revealed an important regulatory role of pro- and anti-inflammatory cytokines in disease progression. These findings have led to the development of promising anticytokine and immunomodulating treatment strategies. We anticipate that ongoing research will expand our knowledge of currently described disease mechanisms and lead to the identification of new pathophysiologic features of sepsis. Also, we expect that novel antisepsis strategies will continue to be clinically assessed and potentially exploited for the more effective future treatment of sepsis.

## Figures and Tables

**Table 1:** Diagnostic criteria for the systemic inflammatory response syndrome (SIRS).

Defined by the presence of two or more of the following clinical findings	
(1) Body temperature >38°C or <36°C	
(2) Heart rate >90 min^−1^	
(3) Respiratory rate >20 min^−1^ or PaCO_2_ <32 mmHg	
(4) White blood cell count >12,000 cells *μ*L^−1^ or <4,000 cells *μ*L^−1^ or >10% immature (band) forms	

Table adapted from [11].

**Table 2:** Summary of the main features of pro- and anti-inflammatory cytokines.

Cytokine	Main sources	Main functions	Interactions with other cytokines	Alteration/involvement in diseases	Physiologic inhibitors and therapeutic targeting strategies	References
Proinflammatory						

TNF-*α*	Immune cells of the innate and adaptive immune system (mainly macrophages and lymphocytes); fibroblasts	Differentiation and activation of immune cells; induction of fever and coagulation; cachexia; apoptosis	Promote the release of downstream proinflammatory effector molecules	Role in atherosclerosis, RA, Alzheimer's disease, autoimmune diseases, and cancer	sTNFRs; anti-TNF Ab; TNFR inhibitors	[35–43]

IL-1	“	Induction of fever and coagulation; hematopoiesis; promotes the extravasation of inflammatory cells	“	Role in autoinflammatory diseases, heart failure, and diabetes	IL-1R2; IL-1Ra; anti-IL-1*β* mAb	[16, 38, 39, 44–48]

IL-6	“	Activation of B and T lymphocytes; modulation of hematopoiesis and acute phase response; induction of fever	Released in response to TNF-*α* and IL-1 but inhibits their release; promotes anti-inflammatory responses (sTNFRs, IL-1Ra, and TGF-*β*)	↑ Serum levels following burns, major surgery, in sepsis, RA, and Crohn's disease	sIL-6R, anti-IL-6 Ab, and anti-IL-6R Ab	[49–63]

IL-12	Monocytes/macrophages; Neutrophils; dendritic cells	Promotes type 1 adaptive immune response and differentiation of T_H_1 T lymphocytes; induces antitumor immune response	Induces IFN-*γ* production	Role in cancer	Anti-IL-12 mAb	[64–67]

IFN-*γ*	NK cells; T_H_1 and CD8^+^ cytotoxic T-cells	Antiviral activity; potentially reverses immunoparalysis in sepsis	Released in response to TNF-*α*, IL-12, and IL-18	↑ Serum levels in sepsis	rIFN-*γ*	[68–73]

MIF	Pituitary cells; monocytes/macrophages	Activation of macrophages and T-cells; overrides the anti-inflammatory effect of glucocorticoids	Released in response to infection, inflammation, and proinflammatory cytokines; promotes the release of proinflammatory effector molecules	↑ Serum levels in acute and chronic inflammatory diseases; role in cancer	Small molecule inhibitors (ISO-1, benzoxazol-2-ones); human anti-MIF Ab; MIF-derived peptide sequences	[69, 74–84]

Anti-inflammatory						

IL-10	Immune cells of the innate and adaptive immune system	Immunosuppressive properties, such as the impairment of antigen presentation and phagocytosis	Suppress the release of proinflammatory cytokines; stimulate production of sTNFRs and IL-1Ra	Dysregulated in autoimmune diseases	rIL-10	[85–88]

TGF-*β*	Macrophages; smooth muscle cells	Involved in tissue repair, fibrosis, and sepsis-induced immunosuppression	“	↑ Serum levels in sepsis; upregulated in cancer and fibrosis	Small molecule inhibitors; anti-TGF-*β* mAb	[89–94]

IL-4	T_H_2 T lymphocytes; mast cells; basophils; eosinophils	Promotes differentiation of T_H_2 T lymphocytes	Induces release of IL-4 and IL-13 from macrophages	Role in scleroderma, asthma, and tuberculosis	Anti-IL-4R*α* mAb	[95, 96]

RA: rheumatoid arthritis; sTNFRs: soluble TNF receptors; mAb: monoclonal antibody; IL-1Ra: IL-1 receptor antagonist; rIFN-*γ*: recombinant IFN-*γ*; ISO-1: (S,R)-3-(4-hydroxyphenyl)-4,5-dihydro-5-isoxazole acetic acid methyl ester.
